# PharmoCo: a graph-based visualization of pharmacogenomic plausibility check reports for clinical decision support systems

**DOI:** 10.1515/jib-2023-0026

**Published:** 2023-12-28

**Authors:** Lena Raupach, Cassandra Königs

**Affiliations:** Faculty of Technology, Bioinformatics/Medical Informatics Department, Bielefeld University, D-33501 Bielefeld, Germany; ID Information und Dokumentation im Gesundheitswesen GmbH & Co. KGaA, D-10115 Berlin, Germany

**Keywords:** pharmacogenomics, pharmacogenetics, clinical decision support system, inter-individual biological variation, data display

## Abstract

The first approaches in recent years for the integration of pharmacogenomic plausibility checks into clinical practice show both a promising improvement in the drug therapy safety, but also difficulties in application. One of the difficulties is the meaningful interpretation of the text-based results by the medical practitioner. We propose here as an appropriate and sensible solution to avoid misunderstandings and to include evidence-based, pharmacogenomic recommendations in prescriptions, which should be the graph-based visualization of the reports. This allows for a plausible interpretation and relate complex, even contradictory guidelines. The improved overview over the pharmacogenomics (PGx) guidelines using the graphical visualization makes the medical practitioner’s choice of dose and medication more patient-specific, improves the treatment outcome and thus, increases the drug therapy safety.

## 1 Introduction

In recent years, research has focused on the variability between inter-individual drug responses, including interactions between drugs and gene variants involved in the drug metabolism [1]. Different variants of the same gene can cause deviations in drug response within a population [2]. The most clinically relevant effects of the resulting polymorphic enzymes are the acceleration or deceleration of drug metabolism [3]. These effects are associated with an adverse drug response or lack of response to medication [4]. For example, if an active ingredient of a drug is decomposed by a *poor metabolizer (PM)*, it remains longer in the blood than usual, increasing the risk of side effects or an overdose. Conversely, being decomposed by an *ultra-rapid metabolizer (UM)* can lead to the loss of the therapeutic effect because of the low blood level of the drug [4].

There are other factors causing the concentration of various metabolites to vary widely even for an individual. Other factors, such as sleep and posture, also affect the elimination of drugs, resulting in different drug levels in the blood [5]. All these factors must be taken into account in combination with the PGx information in order to accurately estimate the actual drug concentration in the blood.

By curating primary literature, institutions like the *Pharmacogenetics and Pharmacogenomics Knowledge Base* (PharmGKB) [6] created a solid, evidence-based database of PGx annotations for individual drug prescription guidelines [6]. The annotations do not only offer information about the velocity of a drug, but also about the toxicity, efficacy, dosage and metabolism in association with certain gene variants. The velocity does not necessarily indicate toxicity, as the example of tramadol and the gene variant CYP2D6*1xN demonstrates. Even if the drug is rapidly metabolized by this gene variant, it can also show toxicity and that can be cumulative [7]. A regular, e.g. daily, administration of a drug that is metabolized more slowly than expected may increase the risk of an overdose. This is due to an approximately exponential rising concentration profile caused by a poor metabolism of the drug. This shows the importance of taking the metabolism velocity of each drug into account.

There already has been studies for preemptive PGx testing, for example in the PG4KDS program of the St Jude Children’s hospital [8] or the Vanderbilt University Medical Center [9]. Though the quality and quantity of PGx research has improved over the last two decades [4], the implementation of PGx knowledge into medical care is still challenging. Three of the barriers for the application of PGx testing in clinical practice are the lack of evidence for utility and efficacy proven by clinical trials, especially for non-European ethnic groups, the lack of proof of cost-effectiveness and lack of knowledge regarding PGx by medical practitioners [4]. The latter issue will be tackled by the visualization, as it helps the medical practitioner with interpreting the warnings reported by a PGx medication check.

At the moment, the prescription of standard doses and thus, the trial-and-error-approach for drug prescription is yet common [10]. With PGx getting increasing focus for academic research, policy makers are increasingly interested in using them in clinical practice [11]. The PGx patient characteristics, for example enzymes that are PMs for a drug because of their genetic predispositions, can effect the drug pathway in a way that impacts the drug efficacy fundamentally [2]. For example, PMs decompose a drug slower that a *normal metabolizer (NM)*, causing a higher blood level of the drug and consequently, an increased risk of side effects or an overdose [2].

Utilizing PGx preemptive testing in clinical practice has already been proven as a promising approach to prevent inadequate prescriptions [8, 12]. A problem that occurs in practice is the mass of PGx information, that must be processed by the medical practitioner in order to integrate all displayed PGx warnings into the decision of the prescription and drug dose [11]. Text-based guidelines take time to be read and even then, having multiple enzymes in the pathway, the guidelines rarely all give the same instructions. The characteristics of the enzymes are independent of each other, so contradictory guidelines within a pathway can occur. An approach is needed that displays all guidelines, including conflicting ones, and illustrates them at a glance. That visualization would be another necessary step towards the successful implementation of PGx in clinical practice, and provide an overview in the flood of PGx information during the prescription. The main target group for this visualisation tool are medical practitioners, as they have a basic knowledge of the molecular properties of drug metabolism and the tool thus represents a supplement to the text-based explanations of the PharmGKB dose recommendations.

For that purpose, we introduce PharmoCo1https://gitlab.ub.uni-bielefeld.de/lraupach/pharmo-co. as a first approach of visualizing text-based PGx guidelines in order to get an overview over complex and potentially contradicting information. The medical practitioner is intended to see the patient’s PGx characteristics for a specific prescribed drug at a glance, so that they can react and, if necessary, adjust the prescription based on the effects of the patient’s gene variants to the drug metabolism.

## 2 Related works

This visualization approach is based on the concepts of the two following related works. The modelling of the PGx results as a network graph was based on PharMeBiNet [13], a database and a website where analysis checks can be executed and visualized. The medication check ID MEDICS^®^ [14] is listed as related work because it provides an existing medication therapy workflow used in hospitals, where the PGx reports would be an addition to increase the safety of the patient’s medication therapy.

**Figure 1:**
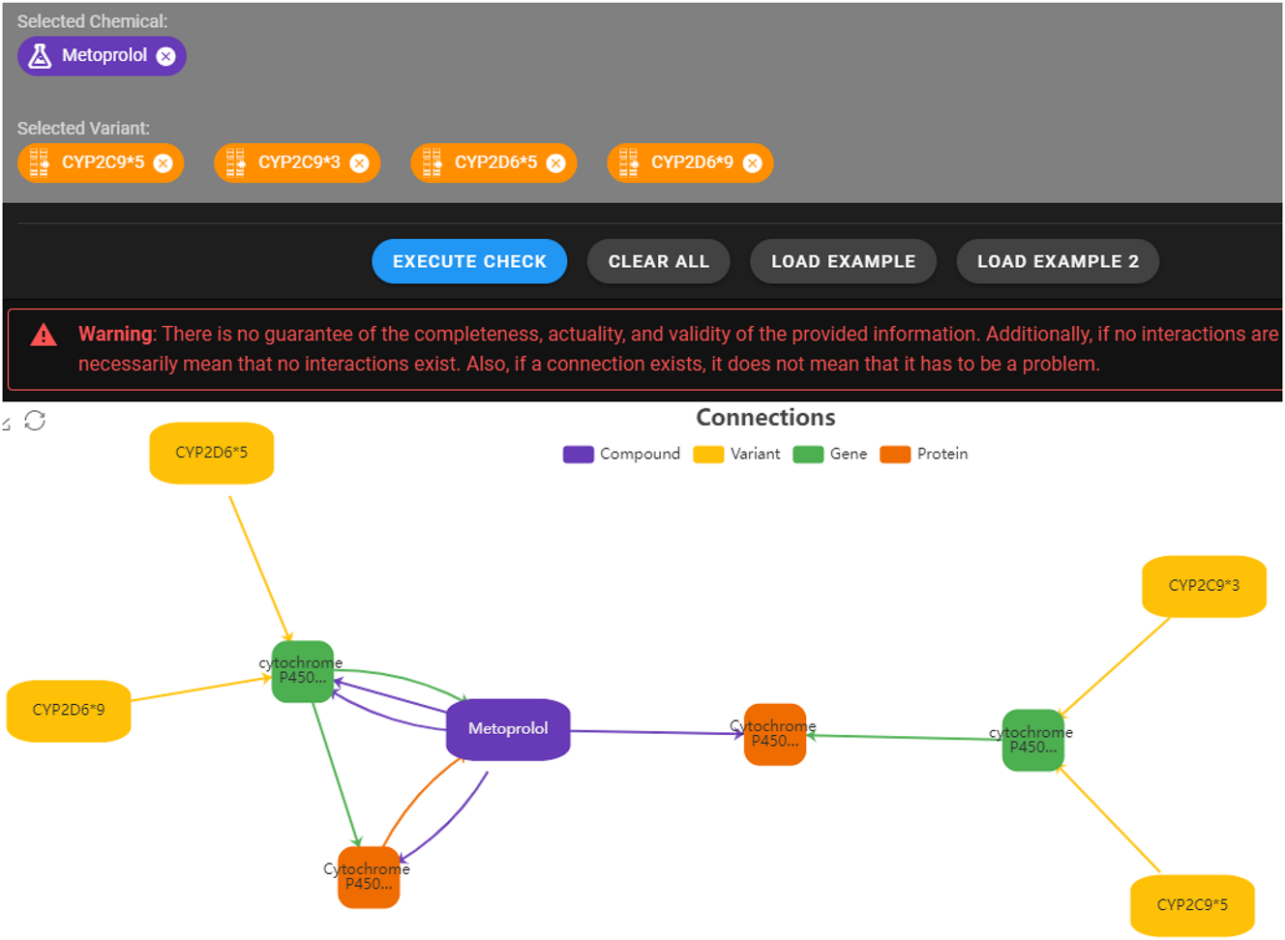
Example of the drug metoprolol and variants CYP2C9*3/*5, CYP2D6*5/*9 of the PharMeBINet analysis of potential drug-gene variant interaction. Genes are represented by the green nodes, the drug metoprolol by the violet node, proteins by orange nodes and the yellow nodes represent the gene variants. The edge colour mirrors the colour of the outgoing node [13].

PharMeBINet [13] is a database that contains information about genes, drugs, proteins, gene variants, pathways, diseases, and other entities. An analysis of potential drug-gene variant interactions can be accessed via the PharMeBINet website [15]. Figure 1 shows an example of a potential drug-gene variant interaction between metoprolol and the variants CYP2C9*3/*5, CYP2D6*5/*9. A connection between entities is constructed if a drug interacts in any way with a protein or gene of the gene variant. The visualization is generated as a network graph using the charting and visualization library *ECharts* [16]. The graph-based visualization gives an overview over molecular interactions and shows in detail, how the gene or protein is associated with the drug. However, this interaction is estimated, not evidence-based. Additionally, no recommendations regarding drug choice or dose adjustment is integrated in this visualization approach, as it contains estimated interactions that might lead to a recommendation for a dose adjustment.

**Figure 2:**
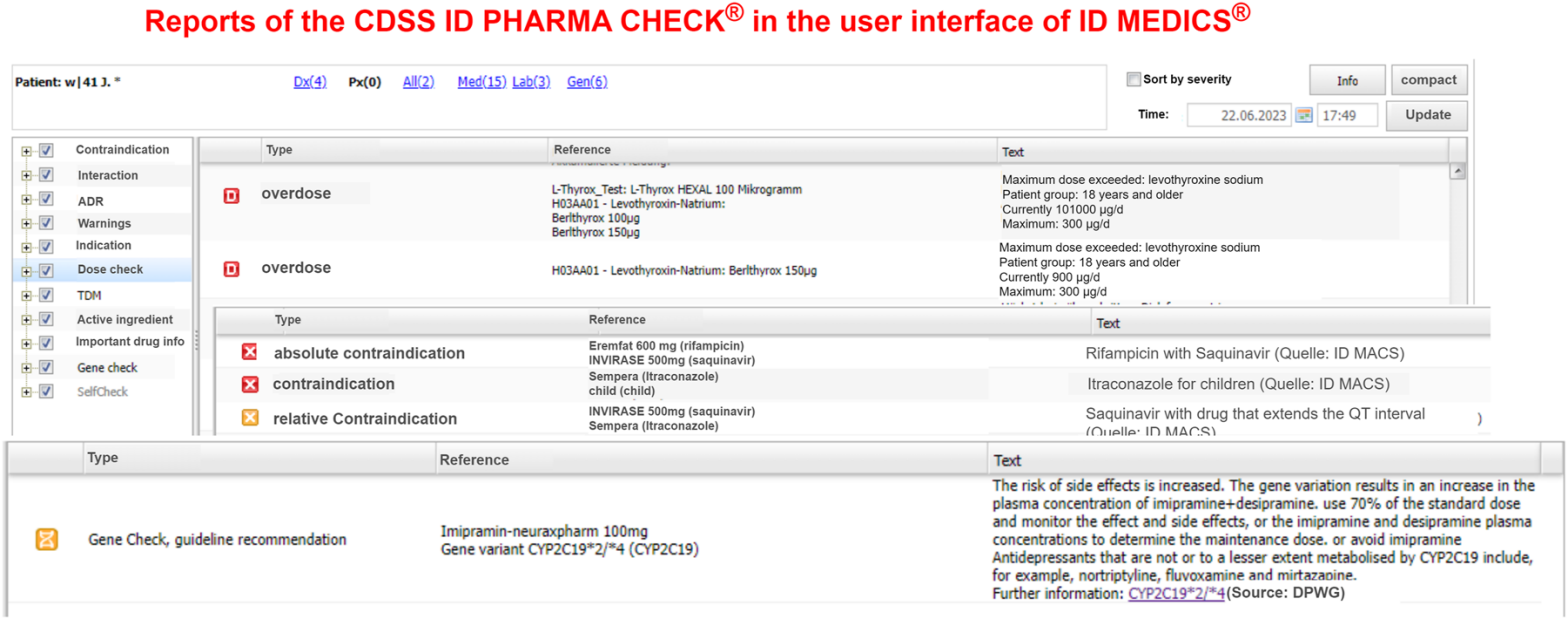
Examples of ID PHARMA CHECK^®^reports in the graphical user interface of ID Medics^®^. In the background, overdose, and contraindication reports are displayed. The front shows a pharmacogenomic check report containing a dose recommendation for the prescribed drug imipramine and the patient’s gene variant CYP2C19*2/*4 [14].

The next related work is ID MEDICS^®^by ID Berlin [17], a software for digital medication management. It is certified as a medical device by the *Medical Device Regultation* and consists of multiple components for anamnese, dosage, medication overview etc. ID MEDICS^®^supports the medical practitioner at clinical decisions using the ID PHARMA CHECK^®^, the drug therapy safety component, checking the prescription i.e. for contraindications or overdoses. It is used in over 1200 hospitals in Germany, Austria and Switzerland and is designed for remedial safe, reliable coding and medication. For this purpose, the ID PHARMA CHECK^®^works as a rule-based AI checking interactions between drugs, but also allergies, and, more recently, drug-gene interactions as PGx check. The ID MEDICS^®^user interface is shown in Figure 2.

The figure shows alerts for different cases by the ID PHARMA CHECK^®^. The two upper tables of Figure 2 show a patient’s medication prescriptions causing multiple overdose and contraindication reports with corresponding explanations in the text column on the right. The front image displays a textual representation of a PGx check report containing a dose recommendation for imipramine based on the gene variant CYP2C19*2/*4[14]. These textual representations of the reports give a comprehensive, but in the case of drug-gene variant interactions, not an easily understandable overview of all the peculiarities of the patient-specific drug pathway. This related work is therefore the predecessor in the visualization of drug-gene variant interactions.

The aim of PharmoCo is to combine these two works. ID MEDICS^®^is able to produce patient specific PGx reports, such as dosage recommendations, based on the patient’s gene variants. These recommendations can be many and sometimes contradict each other. The current report of ID MEDICS^®^are text-based, but these complex information is hard to understand if listed in full texts. In this work, a more descriptive approach to the representation of these reports will be developed. How and where these recommendations are involved in the metabolic pathway of the drug would lead to a better understanding of it. So the main objective of this work is to integrate the complex PGx information of an PGx medication check as ID MEDICS^®^and represent it as a network graph as in PharMeBiNet.

## 3 Methodology

**Figure 3:**
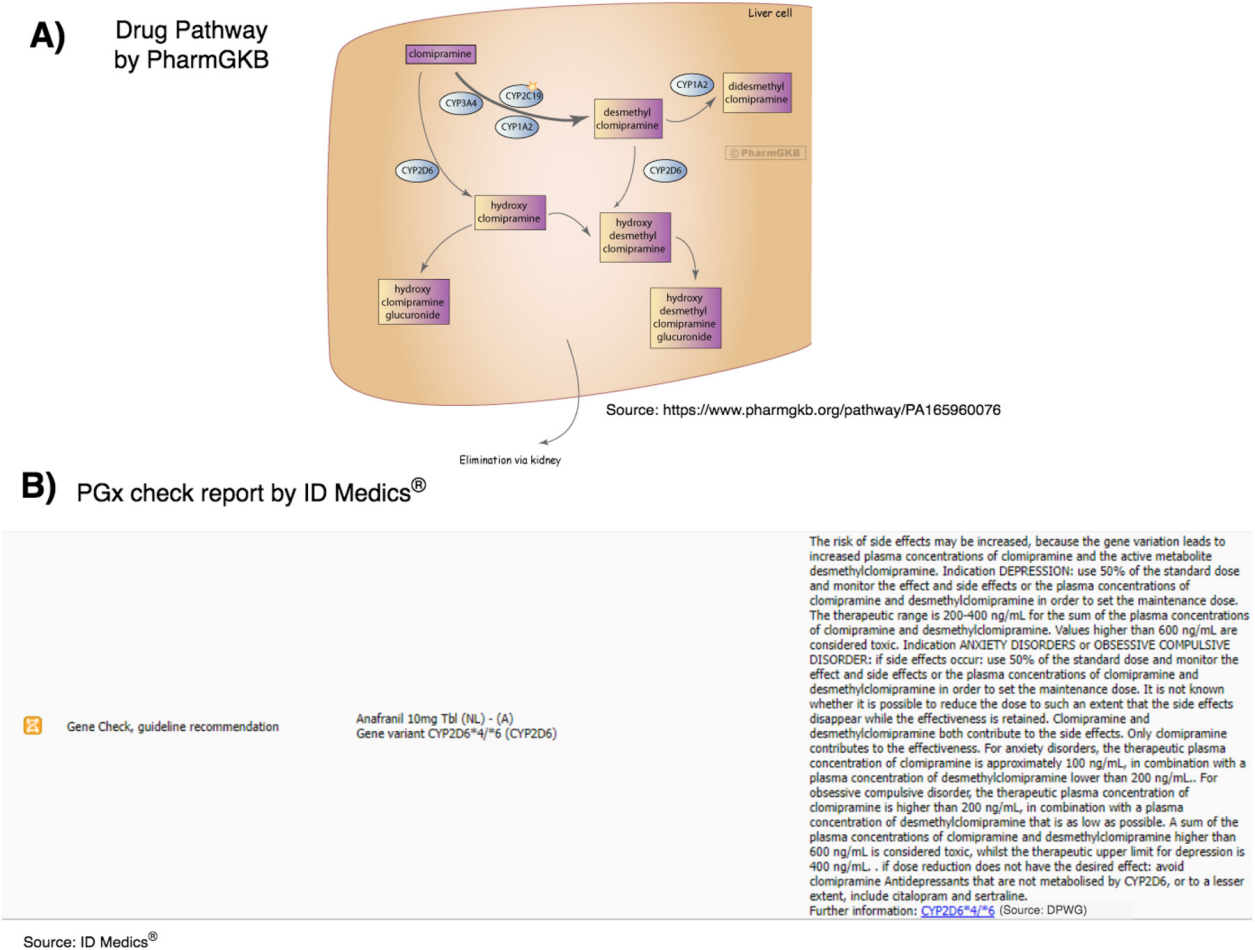
The two input data for the generation of the PGx visualization: (A) the drug pathway of clomipramine by PharmGKB [6]. (B) The text-based PGx report by ID MEDICS^®^of clomipramine and the gene variant CYP2D6*4/*6 containing the dose recommendation text on the right side.

The concept of the visualization of PGx report guidelines is to display the drug metabolism pathway in combination with the patient specific guidelines in a network-graph structure. Shown in Figure 3(A), the metabolism pathway of clomipramine [18] begins in the liver cell. Clomipramine is used as an example prescription, since it has an clearly structured drug metabolism pathway as well as clinical annotaitons in the PharmGKB database. The enzymes CYP3A4, CYP2C19, CYP1A2, and CYP2D6 decompose the initial agent to different metabolites. The metabolization step from clomipramine to desmethyl clomipramine in the upper part of the figure is done by three different enzymes that can all be expressed differently in individuals.

In Figure 3(B), a PGx report from ID MEDICS^®^is displayed. On the left side, the *PGx check* symbol (an orange DNA helix) marks the report with the category “Gene Check” and “guideline recommendation”. The middle part of the figure shows the references to the drug and the gene variant. In this case, anafranil (with clomipramine as active agent) and CYP2D6*4/*6. On the right side, the text represents the guideline warning, in this case from the DPWG data source.

For the concept of the PGx visualization by PharmoCo, drug pathways like in Figure 3(A) should be combined with the information of (B). The nodes are displayed as specific symbols and interactive features in order to visualize the patient specific characteristics in the pathway. In our example, the enzyme CYP2D6 is a PM for clomipramine, meaning that at this point of the metabolization pathway, the speed of metabolization of this drug is decreased compared to the wild type of CYP2D6. This decreased metabolozation speed is marked as such using an arrow down as enzyme symbol. This metabolizer status comes with a guideline that should be made visible to associate it with the respective enzyme node. Beside, a legend for the symbols should explain the used symbols, and a additional, textual representation of the patient data on the edge of the figure is also planned.

## 4 Implementation

The prototype implementation of the visualization called *PharmoCo* is based on a drug pathway file stored in the back-end and an input file containing patient data.Both are used to display the drug pathway and the patient specific PGx information as an interactive network graph to the user.

### 4.1 Input data

The visualization of the PGx reports by PharmoCo shows the drug pathway as a network graph which is described in detail in Section 4.2. Two input files are necessary for the visualization: One file containing the drug’s metabolism pathway and one that stores the patient information and the PGx reports. The pathway files come from PharmGKB (https://www.pharmgkb.org/pathways) and are saved in the backend before runtime. The patient data and PGx reports are integrated into a JavaScript Object Notation (JSON) formatted file and come from an external rule-based clinical decision support system (CDSS) like ID MEDICS^®^. They contain demographic patient data as well as a list of guidelines for each drug. The guidelines consist of the gene variant, it’s activity score, metabolizer status for the expressed enzyme, the drug and it’s Chemical Abstracts Service (CAS) number, the recommendation for action, a severity degree for the urgency of compliance, level of evidence by PharmGKB, the source URL, recommendation directions as categories (i.e. “increase dose” or “avoid”), and a text of the effect of the gene variant on the metabolism. These CDSS rules are based on various data sources, including the dose guidelines and clinical annotations from the PharmGKB and *Dutch Pharmacogenetics Working Group* (DPWG). ID PHARMA CHECK^®^uses the graph-based data warehouse GraphSAW2 [19], that contains external PGx annotations, as a rule base for the PGx check during the prescription. The resulting reports contain dose guidelines and clinical annotations from evidence-based external sources and get exported for further processing in JSON format by the medication management system. When the report is created, the visualization is planned to be made accessible via a button within the text-based report by the ID PHARMA CHECK^®^ *graphical user interface* (GUI).

### 4.2 Implementation of the graph visualization

A web-based application is created for interactivity with the network-graph. The website is built with HTML and JavaScript. Bootstrap v5.3.0 (https://getbootstrap.com) is used for the front end and the framework ECharts v5.4.2 [16] to create the graph visualization. The source code of PharmoCo is available at https://gitlab.ub.uni-bielefeld.de/lraupach/pharmo-co. The visualisation was created in consultation with a pharmacist from ID Berlin [17]. This does not replace a comprehensive evaluation of the tool, but it did provide guidance from the perspective of a medical practitioner during development.

**Figure 4:**
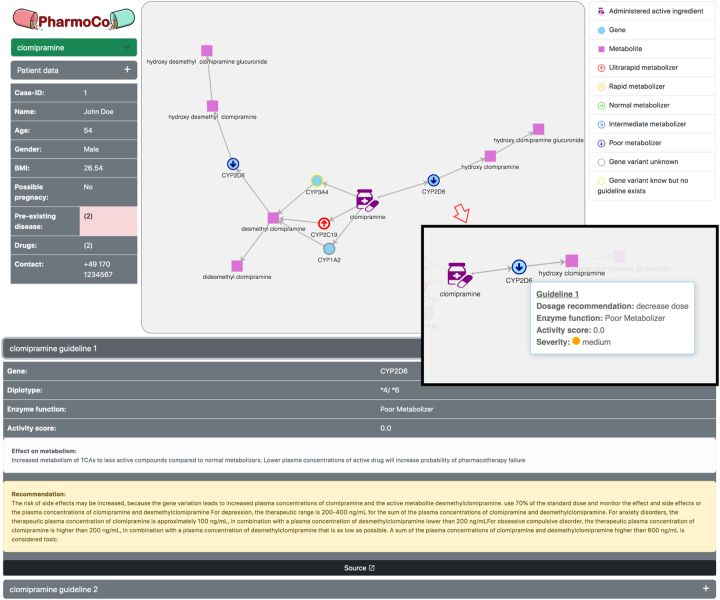
The user interface of PharmoCo is shown using an example of a fictitious, male patient named “John Doe”. The patient was prescribed clomipramine and his gene variants CYP2D6*4/*6, CYP2C19*17/*17 and CYP3A4*1A/*1B are shown in the drug metabolization pathway. The general patient data is displayed on the left. In the middle, the drug pathway of clomipramine is shown as a graph having symbols for the metabolizer status of the respective enzyme node. Right next to it, a tooltip over the blue enzyme node shows a short summary of the corresponding guideline. The guideline details are shown in the bottom part of the figure in as text. The legend on the right side explains the symbols used in the graph.

In Figure 4, the GUI of the visualization website of a fictitious, male patient named John Doe is shown. In the center, the network-graph window displays the drug pathway of the example drug clomipramine. The enzymes have different symbols, depending on their metabolizer status. CYP2C19 stands out having a red upwards arrow as symbol, signalizing that this enzyme is a UM for clomipramine. This means that clomipramine is metabolised faster by this enzyme compared to the wild type. On the other hand, the node of CYP2D6 symbolizes this enzyme as a PM using a blue downwards arrow. By hovering over the enzyme node, a tooltip shows detailed but concise information about the metabolizer status, the guideline recommendation, the enzyme activity score, and the severity of the guideline as indicated in the small window on the right of the graph.

By clicking on the corresponding enzyme or the guideline, the text of the drop-down menu opens and shows the detailed information about the guidelines on the lower part of the figure. There, information such as the guideline texts, the implications and source URL can be accessed.

On the left side of the figure, general patient data like name, age, body mass index, and more are listed which can be useful in decision-making for the drug and dosage. Additionally, information about drugs and diseases is accessible via a tooltip by hovering over the digits that represents their number.

At the upper left part of the page, the PharmoCo logo is displayed. Underneath, a green dropdown list is available, that can be used to choose the drug to be displayed in the graph. On the right side, the legend to the symbols in the graph is provided. A NM would be displayed as a green arrow pointing right and intermediate metabolizers would have a light blue arrow pointing to the lower right. For genes with no metabolizer status available, but with an assigned gene variant in the data, a blue circle with a yellow border is used as the symbol. This case is shown in the center of Figure 4 for the gene CYP3A4.

After choosing the drug using the green dropdown list, the graph visualization is generated at runtime. If a gene appears in multiple metabolizing steps in a single pathway, such as CYP2D6 in the example of Figure 4, the respective gene is displayed multiple times. This prevents the pathway visualization from forming tight clusters but rather to be structured as chains. For a clean structure, the built-in *force layout* is used for the ECharts graph so the nodes repell each other.

## 5 Application

As can be seen in Figure 4, PharmoCo’s GUI shows the individual entities of the drug pathway as nodes and the edges represent the metabolic steps. Symbols, colors, tooltips and text fields are used to integrate the patient data into the graphic. In this example, a fictitious 54-year-old male patient has been diagnosed with both ADHD and depression and therefore, atomoxetine and clomipramine are prescribed, respectively. Three gene variants CYP2D6*4/*6, CYP2C19*17/*17, and CYP3A4*1A/*1B are persisted in his patient file, so that an automatically triggered PGx check from a CDSS can access the prescription as well as the gene variants. The resulting PharmoCo visualization in Figure 4 shows the pathway of clomipramine, whereas the pathway of the second prescribed drug atomoxetine can be accessed via the green drop-down menu in the upper left part of the figure. All three gene variants are visualized in the figure since they play a part in the metabolization pathway of clomipramine. CYP2D6*4/*6 is marked as a PM by the blue downwards arrow corresponding to the guideline at the bottom showing that the enzyme is a PM for clomipramine. The description text “Effect on metabolism” states “Increased metabolism of TCAs to less active compounds compared to NMs; Lower plasma concentrations of active drug will increase probability of pharmacotherapy failure” [20]. Below, the guideline text states that the risk of side effects is increased and recommends to lower the dose to 70 percent of the standard dose. Looking at the other gene variants, the guidelines differ. CYP2C19 is marked as a UM and the guideline recommends the medical practitioner to avoid tertiary amine. So, for the same drug two varying recommendations are displayed in the visualization.

**Figure 5:**
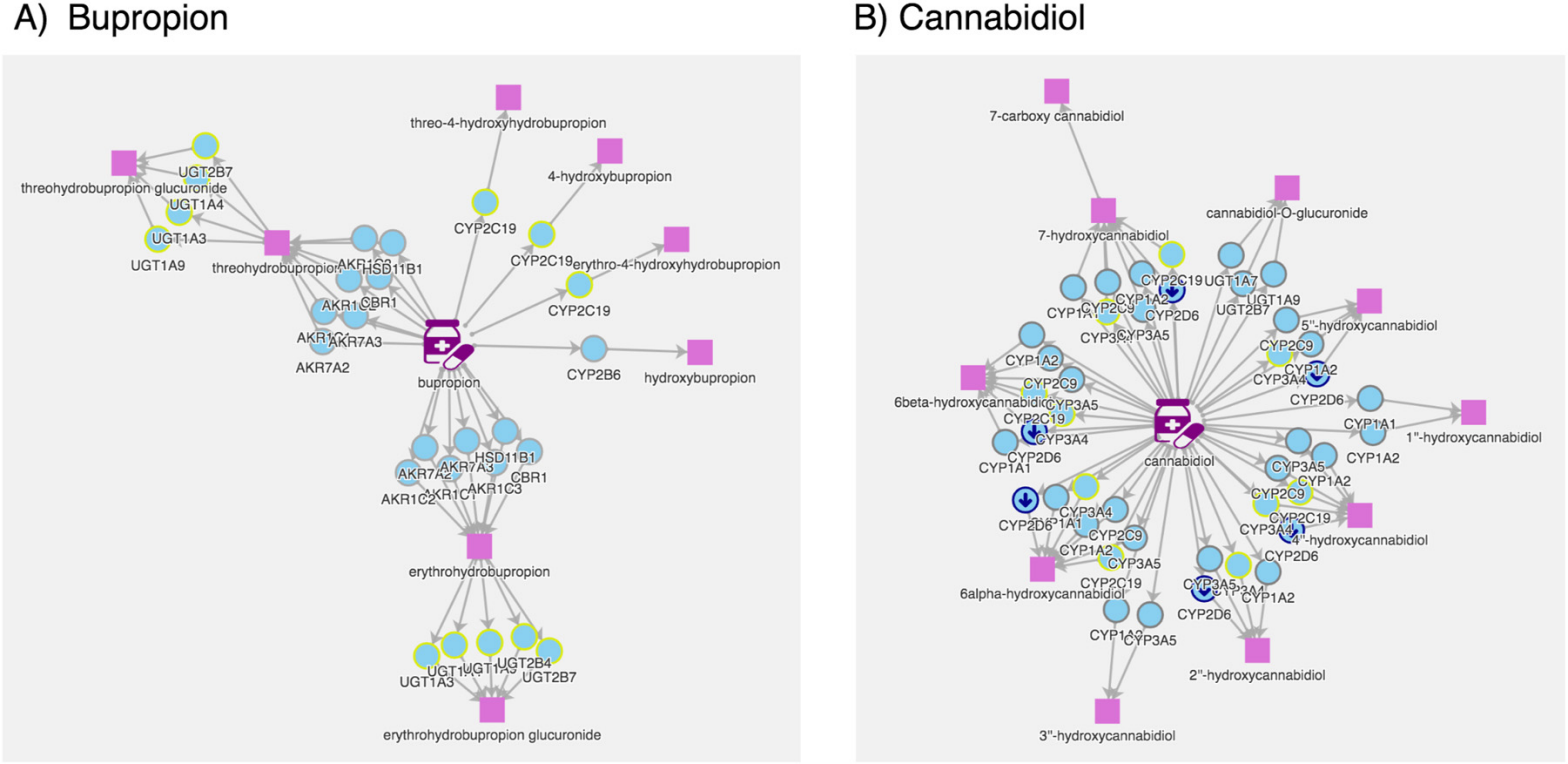
The pathway visualization of PharmoCo with the more complex examples of (A) bupropion and (B) cannabidiol.

Figure 5 visualizes two more complex examples of bupropion on the left side (A) and cannabidiol on the right side (B). The complexity of the graph is higher due to a higher number of metabolites and enzymes.

## 6 Discussion

This approach combines the graph-based visualization concept of PharMeBINet with the texts of the PGx check reports by ID MEDICS^®^containing guideline recommendations. The examples of Section 5 show an overview over complex and potentially contradicting information of the PGx recommendations on a molecular level. The medical practitioner gets to see the patient’s genetic characteristics and patient-specific dose recommendations in a network graph. Using new gene nodes for each reaction, even if they exists on other places in the pathway, leads to duplicated nodes, but also ensures the representation of the pathway as a chain, rather than a tight cluster, resulting in a more readable graph. What stands out most in this graph are the enzyme symbols that indicate the metabolizer status. Thus, in the example of Figure 4 it can be seen at a glance that there are two enzymes in the pathway whose metabolization statuses diverge in different directions. From this it may be concluded that the associated guideline recommendations have two contradictory statements regarding the dose. Hovering over an enzyme, a tooltip shows a short summary of the guideline with the metabolizer status, the recommendation, the activity score and the severity. These information comes from clinical annotation and dose guidelines by PharmGKB. This is the reason why this database is listed as main source of information. These information is available only in text-based files, so GraphSAW2 serves as a intermediate tool to extract, transfer and load them into a requestable database. GraphSAW2 has the advantage of efficiently storing and retrieving the massive, highly interconnected amount of molecular and PGx information in a data warehouse. A medication check such as ID MEDICS^®^can therefore query this data warehouse. As the ID MEDICS^®^software is an existing medication software used in hospitals, it makes sense to integrate the visualisation into this check.

Thus, the medical practitioner is shown key information that may effect decision to a more personalized prescription.

Above a certain amount of metabolites, a pathway may not be able to be visualized clearly by this approach, having too many metabolites and duplicated enzyme nodes in one graph. A possible solution would be the highlighting of duplicate nodes while hovering over one of them contributing to a better overview. Currently, only biochemical reactions of the pathway are considered. But there are more reaction types, such as inhibition, transportation, or complex assembly. In this case, different symbols at the tip of connecting edges would need to be visualized. Another issue that still needs to be addressed are multiple guidelines from different external sources for the same drug-gene pair. In turn, several sources can have different or contradicting guidelines for the same drug-gene pair, which also has to be marked explicitly by the visualization. The quality of the whole visualization is currently highly dependent on the accuracy and schema consistency of the source data since the data integration is automatized. In summary, this approach shows a way to make the usage of PGx check reports by a plausibility check more understandable and thus, paves the way to a more precise drug therapy.

### 6.1 Outlook

It is planned to integrate this visualization into the ID PHARMA CHECK^®^module and analyse the added value to the drug therapy safety in practice. The raw-report is planned to be able to be transmitted via the Fast Healthcare Interoperability Resources (FHIR) standard. An interface definition has to be made for the integration of the raw report to be able to communicate the results with FHIR-based data exchange software. Additionally, it is planned that unaffected reactions by the patients gene variants or reactions where the gene is not available in the source data can be collapsed to further reduce the visual complexity. In the future, the pathway data is planned to be stored in a back-end database having a standardized schema rather than in tab-separated values (TSV) files, because in this text-based file format, the data of PharmGKB do not always follow the same schema. This type of visualization is also interesting for other medication analyses such as contraindication or allergy checks, which is also included in the ID PHARMA CHECK^®^. The information that two drugs are contraindicated to each other can be better understood by showing their molecular pathways in a visualization component, because they overlap at certain points. This would lead to a deeper understanding at the molecular level and thus to a more targeted individual medication process. Patients could possibly benefit from a simplified form of this visualisation, because educating patients of their treatment the patient-specific adjustments could lead to greater patient satisfaction. For a meaningful evaluation of this visualisation tool, a study is to be carried out in the future that includes both medical practitioners and patient opinions. Exact figures on which dose recommendation was used how often compared to the text-based listing of PGx reports will provide information on the benefits of this tool.
